# Concrete Messages Increase Healthy Eating Preferences

**DOI:** 10.3390/ejihpe10020049

**Published:** 2020-06-18

**Authors:** Emily Balcetis, Madhumitha Manivannan, E. Blair Cox

**Affiliations:** Department of Psychology, New York University, New York, NY 10003, USA; mm7314@nyu.edu (M.M.); ec3720@nyu.edu (E.B.C.)

**Keywords:** abstraction, construal level, junk food, temporal distance

## Abstract

Public health campaigns utilize messaging to encourage healthy eating. The present experimental study investigated the impact of three components of health messages on preferences for healthy foods. We exposed 1676 online, American study participants to messages that described the gains associated with eating healthy foods or the costs associated with not eating healthy foods. Messages also manipulated the degree to which they included abstract and concrete language and the temporal distance to foreshadowed outcomes. Analysis of variance statistical tests indicated that concrete rather than abstract language increased the frequency of choosing healthy over unhealthy foods when indicating food preferences. However, manipulations of proximity to outcomes and gain rather than loss frame did not affect food preferences. We discuss implications for effective public health campaigns, and economic and social cognitive theories of persuasion, and our data suggest that describing health outcomes in concrete rather than abstract terms may motivate healthier choices.

## 1. Introduction

The increased prevalence of non-communicable diseases such as cardiovascular disease, diabetes, stroke, and various types of cancers has mobilized a multidisciplinary approach to manage the current health crises [1]. Many of these approaches attempt to modify an individual’s behavior and lifestyle using cognitive-behavioral and social learning theories [2,3], and implement these theories with messages delivered to the public at large urging the adoption of healthy behaviors [4]. The messages are framed in varied ways. Various public health organizations have generated hundreds of thousands of such messages, the specific content and focus of which has varied substantially. For instance, one New York City anti-soda campaign used shame to promote behavior change, presenting glasses overflowing with gelatinous, orange-tinged substances accompanied by the tag line “Don’t drink yourself fat.” A poster in Atlanta attempted to motivate by using fear, depicting an overweight child who could not fit inside the visual frame along with the text, “Warning: Chubby kids may not outlive their parents.” We join the researchers, public health advocates, and community agencies to continue to investigate what forms of messaging—the features of the message frame—promote intentions towards better health. Additionally, we probed the social-cognitive factors that target individuals’ preferences in choosing foods to eat.

### 1.1. Gain- and Loss-Framed Messages

One aspect of public health messages that fundamentally distinguishes one health campaign from another is whether the message focuses on or is framed around gains or losses. The American Cancer Society [5], for instance, recently advocated for gain framing. It highlighted the health improvements that arise after quitting smoking, both immediately such as improved circulation and lung function, and later on such as a reduction in the risk of developing coronary artery disease. In contrast, the Centers for Disease Control [6], has rolled out several ad campaigns that rely on loss framing. Its messages use testimonials from past smokers to encourage current smokers to quit by highlighting what can be lost if a smoker continues, including jaw bones, the ability to speak, and the freedom to move unencumbered by oxygen tanks.

Researchers have examined whether gain or loss message frames are more effective at motivating individuals to engage in healthy behaviors. One meta-analytic review examined the effect size of gain and loss framing aggregating across 94 published studies. They concluded that gain-frames were more effective than loss-frames at motivating healthy behaviors including exercise, smoking cessation, and skin cancer prevention [7]. However, whether gains or losses consistently motivated behavior change is open to discussion. Closer inspection finds that the effect size of the difference in that meta-analysis though significant, was small by all standards (r = 0.083; [7]).

Moreover, the results of another meta-analysis of 53 studies found a small though statistically significant advantage for loss-framed over gain-framed messages, an effect that was similarly small (r = 0.039) but opposite in its prescriptive implications [8]. Likewise, another meta-analytic review found that fear appeals, which encourage people to avoid the negative consequences that failing to exercise might induce [9,10], increased individuals’ adherence to doctor recommendations when compared to exposure to no messages, to a message that was not designed to induce fear, or to messages designed to induce relatively less fear than the experimental group [11].

The efficacy of gain or loss frames may depend on not only the gain or loss frame of the message but other features of its content as well. Both gain-frames and loss-frames can induce compliance with messages advocating for behavior change, however, whether gain or loss-frames are stronger motivators of healthy behavior may depend on additional characteristics of the language the messages contain. With this work, we draw from two theoretical fields—economic models and social cognitive theories—to test the efficacy of gain and loss frames, in addition to their interaction with multiple other message features.

### 1.2. From the Economics of Message Framing

Economic models posit that temporality could interact with the gain and loss frame of a message. Messages intended to change behavior could include descriptions of distant, long-term or proximal, short-term outcomes, and could vary the content of messages through differences in the time units specified (e.g., infection rate per day vs. per year; [12]) or the immediate or delayed nature of the consequences [13]. Evidence suggests that immediate rather than delayed outcomes motivate behavior change. For instance, when researchers stated that drinking holds immediate negative consequences to college students, alcohol use and frequency of binge drinking were lower, compared to when researchers stated that drinking holds long-term negative consequences [13]. Future outcomes may carry less weight and exert less impact than immediate outcomes (e.g., [14,15]), which suggests that messages describing proximal rather than distal consequences could impact health decisions to a greater degree.

However, it is possible that the degree to which proximal or distal frames shape the efficacy of gain or loss frames varies. The hyperbolic discounting function and temporal discounting function, in fact, propose that individuals value gains less as they become more distant in time (e.g., [16,17]). As a result, the healthiness of behaviors should grow stronger when messages focus on the positive outcomes that will be experienced relatively sooner rather than later. Indeed, gain frames were better than loss frames at motivating individuals to quit smoking and consume less alcohol, and this effect was most pronounced when the gain frame featured immediate benefits over distant future ones [13,18].

The economic principle of loss aversion, however, predicts a larger impact of short-term losses than short-term gains. When researchers incentivized smokers to quit, participants were more likely to achieve at least one day of abstinence after exposure to loss rather than gain frames [19]. When focusing on the immediate, proximal, daily outcomes, loss-framed messages induced stronger and healthier behavior change than gain-framed messages. Together, economic principles specify that temporally near outcomes, whether described as gains or losses, should motivate behavior change more strongly than messages that focus on distal outcomes.

### 1.3. From Social Cognitive Theories of Message Framing

We also draw from social cognitive theories, including Construal Level Theory [20], to generate hypotheses regarding the impact of message frames on health outcomes. Construal Level Theory states that events, including high-stakes decisions and personal experiences involving individuals’ health, can be represented in either high-level or low-level ways [21]. There are multiple ways to induce high and low level construals in message frames, which might impact health behaviors.

#### 1.3.1. Temporal Distance

High and low-level mindsets can be induced by temporality [20,22,23]. High-level mindsets arise when considering long-term implications, while low-level mindsets arise when considering proximal implications. When the psychological distance matches the gain and loss frame, persuasive ability increases. For instance, Canadian residents who viewed advertisements that saw the headline, “Recycle for a better Calgary Tomorrow,” and considered what would be gained by recycling showed stronger pro-environmental intentions than those who read about what would be lost. Similarly, Canadian residents who read the headline, “Recycle for a better Calgary Today,” and considered what would be lost by not recycling strengthened intentions compared to those who considered what could be gained [24]. In other words, when the message paired a distal frame with gains or proximal frame with losses, behavioral intentions grew stronger than when the pairings were mismatched.

#### 1.3.2. Abstraction

High and low-level mindsets can also be induced with abstract and concrete language [20,22,23]. High-level mindsets arise with abstract language, while low-level mindsets arise with concrete language. Evidence suggests that concreteness, associated with low-level construals, rather than abstraction, is associated with persuasive appeal. Nisbett and Ross [25] (p. 44), in fact, theorized that concrete rather than abstract messages make information more available in memory, increases attention to and elaboration and rehearsal of the message, and increases the effectiveness of persuasive appeals, arguing that “people’s inferences and behavior are more influenced by vivid, concrete information than by pallid and abstract propositions.” Indeed, messages describing the risks of sexually transmitted disease and skin cancer is more persuasive when they contain concrete vivid descriptions, like personal stories, specific details, and photographs of consequences, rather than non-vivid descriptions, like general statistics or text [26]. Graphic images of oral disease associated with smoking elicited greater intentions to quit smoking than non-pictorial warnings or less graphic images [27]. And when the consequences of drinking alcohol on reaction time were described in more concrete terms (as resulting in bloody, bone-crushing accidents) rather than abstract terms (as resulting in delays that slow reaction time to a snail’s pace), participants remembered more of the message content [28]. In other words, when people believe they can make necessary changes to reduce risk in their daily life, concrete rather than abstract information produces cognitive and behavioral intention changes that promote good health (see [29], for a review of nuances in how to establish concreteness without confounding variables).

Though there is evidence of an overall motivating effect of concrete rather than abstract language, Construal Level Theory offers that gain and loss frames might be differentially persuasive when described in concrete or abstract terms, as gains align with high-level construals, while losses align with low-level construals [30]. When individuals considered, for instance, what could be lost by a behavior, they engaged a more concrete mindset, while consideration of what could be gained engaged a more abstract mindset, and when messages matched the gain frame with abstraction and the loss frame with concreteness, persuasion increased [24]. These data suggested that matched alignment between construal level and gain or loss frame can increase the fluency of message processing and as a result increase the persuasive appeal of the message content.

### 1.4. Unpacking the Covariation

There are challenges to interpreting existing evidence of the impact of abstraction, temporality, and gain and loss frames in health message frames given covariation in variables of interest. For instance, manipulations of temporal distance and gains and losses naturally give rise to differences in the abstract nature of cognition. Indeed, Chandran and Menon ([12]; Study 3) found that individuals perceived risks more vividly and clearly when messages described the deleterious consequences of failing to exercise when they were ones described as having an impact in one day rather than one year. That is, proximal loss consideration primed a concrete, low-level mindset.

Moreover, some messages that highlight the temporal nature of outcomes simultaneously varied the abstract nature of the language used within them. For example, one investigation varied the temporal context of the message by emphasizing either the immediate or the long-term effects of consuming fruits, but in doing so, simultaneously varied the certainty of experiencing the stated effects [31]. Certainty is a form of abstraction [32]; thus, temporality covaried with abstraction. Such covariation pose challenges to understanding the cognitive processes by which message frames impact behavioral intentions. Given the relationships among time and abstraction, isolating the unique impact of temporal distance, abstract language, and gain or loss frames has flummoxed empirical investigations thus far.

### 1.5. Aims

The current research sought to clarify the nature of message frames that are most persuasive at motivating healthy food preferences, as healthy eating has been shown to have myriad preventative and protective benefits against chronic, preventable disease including heart disease, stroke, and diabetes [33]. The present research used an experimental social-cognitive approach to test the effectiveness of gain and loss-frames under conditions that varied either the abstract nature of the language used to describe them or the temporal distance to described consequences.

We designed a high-powered study in which we manipulated gain and loss frame. We crossed this manipulation with two additional constructs: construal type and level. Construals can be manipulated via changes in abstraction or in temporality. Each of these types of construal can assume low or high levels. When manipulating abstraction, concrete messages reflect low level framing while abstract messages reflect high level framing. When manipulating temporality, proximal consequences reflect low level framing while distal consequences reflect high level framing. Probing the facets of health message framings that are most effective at motivating behavior change will aid the public health community in formulating compelling campaigns aimed at prevention of chronic, non-communicable diseases.

### 1.6. Hypotheses

Given the covariation in manipulated constructs in published research, we posit as an exploratory prediction whether either low or high levels of framing when applied to the dimension of temporality or the dimension of abstraction will predict divergent effects of gain and loss frames on healthy eating intentions.

## 2. Materials and Methods

### 2.1. Ethics

The procedures were reviewed and approved by the New York University, University Committee on Activities Involving Human Subjects, #2016-1090 date: 2-11-2019.

### 2.2. Participants

We recruited 1676 participants from Amazon Mechanical Turk. Participants were eligible to complete the study if they lived in the United States. Respondents were paid $1.00 for completing the survey. We conducted a power analysis using G* Power [34,35] to compute the sample size needed to detect the smallest effect size we were probing, using a priori planned two-way analyses that would deconstruct a 3-way interaction. Because there is no published research we could draw from for the model we designed, we aimed to achieve 80% power to detect a small to medium effect size Cohen’s *f* of 0.175, with an alpha error probability of 0.05, in an interaction with a numerator *df* of 1 and 4 groups, the analysis required 259 participants for any decomposed two-way interactions. Because we designed a study in which there were six possible two-way interactions, we set as our recruitment goal a minimum of 1500 participants. We oversampled in anticipation of attrition and to ensure adequate power; 31 responses were excluded from analysis for failing attention checks and supplying nonsensical answers to open-ended questions that suggested they were bots, leaving data from 1644 respondents for analysis (See Table 1 for participant demographics; data file indicates in "exclude_from analyses" column which participants were removed).

### 2.3. Message Frames

All participants read one of 16 different messages that framed the United States’ growing public health concern with the obesity epidemic using different language. Every message began by stating that countless longitudinal studies have found a strong link between obesity and the development of chronic, non-communicable diseases such as heart disease, stroke, diabetes, and cancer, which are the biggest killers in developed nations.

The message went on to describe recommendations by one of two sets of government agencies, as a manipulation of Government Agency. In one, the message described the United States Department of Agriculture and Health and Human Services daily minimum number of serving of fruits, vegetables, lean meat, and whole grains. In another, the message described The Centers for Disease Control recommendation for adults to eat a variety of healthy, nutrient-dense foods across all food groups and set a calorie level that will help each individual achieve and maintain a healthy body. We selected these two federal agencies that offer compatible recommendations but that vary in the manner in which they describe these recommendations.

The next paragraphs varied the gain or loss frame. Messages were framed as either gains or losses. Among those receiving gain-framed messages, participants learned that engaging in healthy eating would provide health benefits. Among those receiving loss-framed messages, participants learned that not engaging in healthy eating would lead to health risks (See Supplement for exact wording of all messages).

These gain or loss frames simultaneously varied with the type of construal. Some messages held abstraction constant, and varied the temporal distance to the expected consequences. Those assigned to the proximal frame read that the consequences of [not] eating healthy would impact them within weeks (gain/weeks n = 202, loss/weeks n = 202). Others assigned to the distal frame read that the consequences would impact them in years (gain/years n = 211, loss/years n = 201). 

While half of the participants were exposed to variation in temporal distance, the other half of participants were exposed to variations in the abstract nature of the language used in the message, holding temporal distance constant. Some read messages that described the health consequences in concrete terms (gain/concrete n = 212, loss/concrete n = 213). For example, the concrete gain frame contended that adopting and maintaining a regular healthy eating plan will improve the heart’s functioning, allowing it to effectively pump blood throughout the body. The concrete loss frame stated that failing to adopt and maintain a regular healthy eating plan will diminish the heart’s functioning, preventing it from effectively pumping blood throughout the body. Others read messages that described the consequences described in abstract terms (gain/abstract n = 196, loss/abstract n = 207). The abstract gain frame stated that adopting and maintaining a regular healthy eating plan will improve cardiovascular health. The abstract loss frame stated that not adopting and maintaining a regular healthy eating plan will diminish cardiovascular health. In these manipulations, the only mention of time was that the consequences would occur sometime in the future.

To check the manipulation of language abstractions, we submitted our messages to a Coh-Metrix analysis. Coh-Metrix evaluates texts based on input from multiple lexicons including Celex [36], WordNet [37], the MRC Psycholinguistic Database [38], and others. It computes over 700 indices [39]; we focused our analysis on the percentile estimate of the concreteness dimension of the “text easability” principal component scores. Higher scores indicate more concrete words used in the text. Table 2 provides evidence that the text we used in the concrete messages included more concrete language than the text we used in the abstract messages. Within manipulations of temporal distance, the language we used included relatively equal numbers of concrete words regardless of gain or loss framing or distal rather than proximal consequences.

### 2.4. Procedures

After consenting to participate, participants randomly received one of 16 health messages in the 2(Gain, Loss) × 2(Construal Type: Abstraction, Temporality) × 2(Level: High, Low) × 2(Government Agency: USDA + HHS, CDC) between subject design. We set a restriction that participants could not advance the message before 18-s expired. We also set the survey such that the back button was disabled, ensuring participants could only view the message once before answering our primary questions of interest and could not retake the survey as the IP address was recorded and repeat completions were prohibited. We also recorded the amount of time participants spent reading the message.

As manipulation checks, participants used a 5-point Likert scale (1 = strongly agree, 5 = strongly disagree) to respond to statements that assessed whether the message was gain or loss-framed: this message makes me focus on the benefits I would gain by eating healthy; this message makes me focus on what I would lose by not exercising. We subtracted responses to the gain statement from the loss statement to create one gain focus index. A positive score indicated a stronger gain than loss focus, while a negative score indicated a stronger loss than gain focus. To assess temporality, participants read that some points in time feel quite close or quite far away, and then indicated when they expected they would experience the effects of (not) engaging in healthy eating (1 = Very soon from now to 5 = Very far from now).

Following the manipulation check questions, we assessed our primary outcomes of interest. We measured intentions to eat healthy foods. We presented participants with 11 pairs of food items and asked which one they would choose as a snack after having read the message. The pairs included one healthy snack and one unhealthy snack. The pairs were selected to be equivalent in terms of visual features. For instance, an orange bag of chips was juxtaposed against an orange bag of baby carrots. A red bag of chips was juxtaposed against a red apple. A long, brown candy bar was juxtaposed against long, brown pretzel sticks. We computed the percent of healthy foods chosen as our primary outcome of interest. Participants ended by reporting demographics including gender, race, age, and height and weight from which we computed BMI. Data and food pair images are available on OSF: https://osf.io/wv9rf/.

## 3. Results

### 3.1. Time Spent Reading

Because some people spent quite a long time reading the health message (*n* = 9), durations that exceeded 3SDs from the grand mean were replaced with the next highest duration. We conducted an ANOVA predicting time spent reading, with outliers replaced, from Construal Type, Level, and Gain-Loss Frame. The only effect that emerged was a main effect of Construal Type, F(1, 1636) = 5.26, *p* = 0.022, η_p_^2^ = 0.003. People spent 47.5 s (SD = 41.1) reading the message when abstraction was held constant but temporality was manipulated. People spent 52.9 s reading (SD = 51.6) when temporality was held constant but the abstract nature of the language was manipulated.

### 3.2. Manipulation Checks

We ran an ANOVA with Gain-Loss Frame, Construal Type, Level, and Government Agency as predictors of the gain focus index manipulation check. We found a main effect of Gain-Loss Frame, *F*(1, 1627) = 754.92, *p* < 0.001, η_p_^2^ = 0.317. The gain framed message elicited stronger gain than loss focus (*M* = 1.19, SD = 1.62) compared to the loss framed message (*M* = −1.23, SD = 1.94). We also found that Gain-Loss Frame interacted with Level, *F*(1, 1627) = 7.74, *p* = 0.005, η_p_^2^ = 0.005, such that high level (abstract and distant) gain messages elicited a weaker gain focus index (*M* = 1.02, SD = 1.64) than low level (concrete and proximal) gain messages (*M* = 1.36, SD = 1.58), *t*(1627) = 2.74, *p* = 0.006, *d* = 0.211. Likewise, high level loss messages elicited relatively weaker loss focus (*M* = -1.15, SD = 1.92) than low level loss messages (*M* = −1.30, SD = 1.96), *t*(1627) = 1.21, *p* = 0.226, *d* = 0.077, though this simple effect did not reach significance. There was also a weak interaction between Construal Type and Level that we consider spurious and inconsequential for this manipulation check, given that there was no interaction with gain or loss frame, *F*(1, 1628) = 3.83, *p* = 0.05, η_p_^2^ = 0.002.

We ran an ANOVA predicting the time at which they expected to feel the consequences of eating or not eating healthy from Construal Type, Level, Gain-Loss Frame, and Government Agency. We found a main effect of Construal Type, *F*(1, 1628) = 8.95, *p* = 0.003, η_p_^2^ = 0.005, a main effect of Level, *F*(1, 1628) = 35.65, *p* < 0.001, η_p_^2^ = 0.021, a main effect of Gain-Loss Frame, *F*(1, 1628) = 5.40, *p* = 0.020, η_p_^2^ = 0.003, and as expected, an interaction between Construal Type and Level, *F*(1, 1628) = 24.20, *p* < 0.001, η_p_^2^ = 0.015. When manipulating the abstract nature of the message’s language, we held the timing of when participants might experience consequences constant; as such participants in the high-level, abstract language condition did not differ in their expectations of when they would experience health consequences (*M* = 2.84, SD = 0.90) from participants in the low-level concrete language condition (*M* = 2.79, SD = 0.99), *t*(1628) = 0.75, *p* = 0.453, *d* = 0.053. However, participants assigned to the high-level distal, years condition expected they would experience health consequences farther into the future (*M* = 2.93, SD = 0.99) than did participants in the low-level, proximal, weeks condition (*M* = 2.41, SD = 0.95), *t*(1628) = 7.77, *p* < 0.001, *d* = 0.536.

There was also an interaction among Construal Type, Level, and Gain-Loss Frame, *F*(1, 1628) = 4.14, *p* = 0.042, η_p_^2^ = 0.003, suggesting that the gain and loss frame affected the nature of this interaction, but given the weak size of the interaction we do not interpret it here; we report means in the Supplement (see Table S1).

### 3.3. Primary Analysis

We ran an ANOVA predicting the percent of healthy foods chosen from Construal Type, Level, Gain-Loss Frame, and Government Agency. We found an interaction between Construal Type and Level, *F*(1,1628)= 7.15, *p* = 0.008, η_p_^2^ = 0.004. This interaction remained significant even when BMI, age, and gender were included as covariates, *F*(1,1602)= 6.88, *p* = 0.009, η_p_^2^ = 0.004 (Tables S1 and S2). To unpack this interaction, we first looked at preferences among people assigned to the abstraction construal type. Here, temporal distance was held constant. People who read a low-level message that concretely described the consequences of engaging or not engaging in healthy eating selected healthy foods 74.1% of the time (SD = 24.74), while people who read a high-level message that abstractly described the consequences selected healthy foods only 69.9% of the time (SD = 23.87), *t*(1628) = 2.53, *p* = 0.012, *d* = 0.173. Concrete, rather than abstract, descriptions of the impact of healthy eating increased the likelihood of choosing healthy foods.

We next looked at preferences among people assigned to the temporality construal type. Here, abstraction was held constant. We found that temporality did not affect healthy eating choices. People who read messages describing the long-term consequences of healthy diet selected healthy foods 73.9% of the time (SD = 24.14), which did not differ from people thinking about proximal consequences who selected healthy foods 71.8% of the time (SD = 23.88), *t*(1628) = 1.26, *p* = 0.208, *d* = 0.087.

Furthermore, the interaction between Construal Type and Level was not moderated by Gain-Loss Frame, *F*(1,1628) = 1.61, *p* = 0.205, η_p_^2^ = 0.001, or by Government Agency, *F*(1,1628) = 0.87, *p* = 0.350, η_p_^2^ = 0.001. Gain and loss frames did not shift the overall motivating effect of concrete language on healthier food preferences compared to abstract language. Moreover, gain and loss frames were no more motivating when described as either eliciting distal or proximal consequences.

Though we did find an interaction among Construal Type, Gain-Loss Frame, and Government Agency, *F*(1,1628) = 6.11, *p* = 0.014, η_p_^2^ = 0.004, which held even when including gender, age, and BMI as covariates, *F*(1,1602) = 6.03, *p* = 0.014, η_p_^2^ = 0.004, we consider this spurious as it is not moderated by Level which would be required to test possible predictions generated from economic and social cognitive theories. We report the results of this interaction and present the means in the Supplement (see Table S3) in addition to all other results from this model, which were non-significant.

## 4. Discussion

How can health messages be structured to best strengthen intentions to eat healthy foods? In this study, we experimentally tested various facets of message frames and found evidence of a main effect of concrete rather than abstract language. Regardless of whether the message focused on the benefits of eating healthy foods or the costs of not, when consequences were described in vivid, detailed language individuals indicated stronger preferences for healthy rather than unhealthy foods. Moreover, when the abstract nature of the language used was held constant at an average level, discussion of the immediate or long-term consequences of eating habits did not differentially impact reported food preferences.

### 4.1. Null Effect of Temporality

We found that abstraction but not temporality affected healthy food preferences. This result is concordant with other research. For instance, Bernstein, Wood, and Erickson [40] held constant the abstract nature of the language used and manipulated the timing of the possible consequences. Like the results of our study, they too found that the manipulations of short-term and long-term effects of drinking produced no difference in alcohol consumption, within either the gain or loss framed messages. However, our results are discordant with other work. For instance, gain-framed messages strengthened intentions to quit smoking more so than loss-framed messages when warning labels concerned short-term outcomes [18].

Why are the effects of temporality on shifting the impact of gain and loss frames apparently muddled? Temporality, as a means to varying the message frames when attempting to persuade healthy intentions, may be a multifaceted construct that when manipulated or measured taps into more than considerations of time. For instance, temporality can covary with level of construal (see [41] for a discussion). Indeed, when manipulations discuss immediate consequences, the certainty, vividness, and concreteness of the here and now can give rise to a low-level mindset, while the vagaries of the future can induce a high-level mindset. And when temporality and mindset are confounded, timing may appear to shift the effectiveness of gain and loss frames. Whether these results are due to the temporal frame of the message or construal level it induces are unclear. However, when temporality and mindset are disentangled, as they were in this study, evidence emerges that temporality does not shift the relative impact of gain and loss frame.

Moreover, while on the surface it might seem that time and abstraction are two means to inducing a high-level construal—and as a result, should produce similar outcomes—they are not actually interchangeable in this way; abstraction is a direct manipulation of construal level, while temporality is not, necessarily. Psychological distance, including distance induced through temporality, and construal level are separable constructs [22]. While people might find advantages to representing far off things, people, or events in more abstract, high-level ways, they do not by necessity engage that level of representation by simply invoking greater temporal distance.

When this distinction is applied to the context of health messaging, and when researchers aim to isolate the impact of abstraction and temporality, we may be better positioned to craft effective health communications. Indeed, the implications of our results are that manipulations of the abstract nature of gain and loss descriptions may more effectively shift health intentions than do manipulations of the temporal nature of consequences.

### 4.2. Individual Differences in Responding to Health Messages

Additionally, it is important to recognize that health message frames, even those we found to effectively strengthen behavioral health intentions, may not improve outcomes for all individuals. Indeed, past research has shown that individual differences in need for cognition, consideration of future consequences, temporal orientation, and regulatory focus may moderate the effectiveness of gain and loss frames (See [31,42,43,44]). For instance, promotion-oriented people are more influenced by gains, and so abstract, gain-framed messages may be especially persuasive in changing their health behaviors. Prevention-focused people are more influenced by losses, thus concrete, loss-framed messages may be better suited for these individuals. The public health implications of this nuance may then be that campaigns include mention of both abstract gains and concrete losses in a single message. Such a message may be specifically tailored to include content that appeals to the concerns of individuals with varying personality profiles. While our goal was to experimentally isolate the impact of abstraction, temporality, and gain or loss framing, future research may build upon our results to couple those message features we found to be particular effective to probe whether such a message appeals to a wider audience, including those unique considerations of any individual, regardless of chronic regulatory focus for instance.

### 4.3. Domain Specificity

As research on the effectiveness of message frames grows, it may be important to model not only features of the message but also the domain. We focused our investigation on health decisions about which individuals had personal control and that require sustained commitment over time. That is, any single consumption decision will not hurt nor aid goals for good health, and individuals have a high degree of personal control over what they choose to eat. These features of the domain to which the messages applied may also be key determinants of which facet of a message motivate change for the better. In fact, when these features are shifted, so too might the impact of concreteness on beneficial outcomes. Evidence suggests that in cases where an individual is not personally responsible for outcomes, high level construals may be more effective. For instance, school principals who set abstract, future-oriented goals shared new opportunities and the plan for education success for the school and teachers in it more often which increased teachers’ beliefs that they could educate students effectively [45]; abstract and distally oriented mindsets motivated principals to engage in behaviors that improved the experience of students and teachers. Likewise, abstract rather than concrete mindsets increased people’s willingness to perform socially desirable and difficult tasks that benefit others [46].

### 4.4. Strengths, Limitations, and Future Directions

A strength of our approach lies in its experimental quality. Participant-level variables, like education, literacy, language abilities, and other characteristics might affect responding to public health campaigns that are presented to populations at large, and as a result confound attempts to establish the casual effect of components of messages on health outcomes. Though we did not measure individual differences in education, for example, we can still make claims about the causal effect of facets of the messages we presented as these messages were randomly assigned to participants and the effect of any participant-level demographics, for instance, contributes to variance in the data but does not stand as alternative explanations to the conclusions we draw about the impact of concrete language on promoting healthy food preferences.

Of course, we did not measure actual consumption. Actual behavior may deviate from stated preferences [47], so future research should investigate whether concrete messages similarly increase actual food selection and consumption, in addition to preferences.

## 5. Conclusions

More than 7 out of every 10 American adults is overweight [48]. While the reasons are many, one prominent factor is failure to eat a healthy diet. Only 1 in 10 adults meet the Centers for Disease Control’s (CDC’s) daily recommended servings of fruits and vegetables [49]. The current research adds understanding of the social cognitive mechanisms that serve as foundational research for public health programs, including the importance of concreteness in messages such as these, and the many more that will be built in attempts to address the health epidemic seen in the United States and elsewhere.

## Figures and Tables

**Table 1:** Participant demographics.

M_BMI_ (SD)	27.7 (7.6)
M_age_ (SD)	38.3 (12.5)
n White	1268
n Black	149
n Latinx	76
n Asian	68
n Native American, Alaskan, Pacific Islander	13
n other or multiracial	65
n undisclosed race	5

**Table 2:** Percentile estimates of the concrete nature of the language included in each health message when using consequences stated by the United States Department of Agriculture (USDA) and Health and Human Services (HHS) in addition to the Centers for Disease Control (CDC), gathered from Coh-Metrix analysis, as a function of each Abstraction or Temporal Distance manipulation and Gain or Loss frame. Values in parentheses reflect the concrete nature of the unique portion of the text within each government agency manipulation.

	Abstraction		Temporal Distance	
	Abstract	Concrete	Distal	Proximal
USDA/HHS				
Gain	91.47 (85.77)	95.15 (98.84)	86.21 (70.54)	86.21 (70.88)
Loss	92.65 (88.88)	95.54 (96.99)	88.49 (77.34)	88.49 (77.04)
Average	92.06 (87.33)	95.35 (97.92)	87.35 (73.94)	87.35 (73.96)
CDC				
Gain	79.95 (85.77)	90.99 (98.84)	73.24 (70.54)	73.24 (70.88)
Loss	80.78 (86.21)	91.31 (98.96)	78.23 (78.23)	78.23 (77.94)
Average	80.37 (85.99)	91.15 (98.90)	75.74 (79.39)	75.74 (75.41)

## Supplementary Materials

The following are available online at https://www.mdpi.com/2254-9625/10/2/49/s1, Table S1: mean reported perceived distance to when health consequence will be experienced, Table S2: mean percent of healthy foods chosen when BMI, age and gender are not included, Table S3: model parameters when predicting percent of healthy options chosen in a model excluding covariates, Table S4: model parameters when predicting percent of healthy options chosen in a model including covariates of age, gender, and BMI.
