# Supplementary material for: Concrete Messages Increase Healthy Eating Preferences
Source: Eur J Investig Health Psychol Educ. 2020 Jun 18;10(2):669–81. doi: 10.3390/ejihpe10020049 (PMC8314282; doi:10.3390/ejihpe10020049)
Supplement: Supplementary file 1 [file ejihpe-10-00049-s001.pdf]

MESSAGE FRAMES

GOVERNMENT AGENCY: USDA AND HHS

Gain Concrete

The United States currently faces a growing public health concern with the obesity epidemic. Countless longitudinal studies have found a strong link between obesity and the development of chronic, non-communicable diseases such as heart disease, stroke, diabetes, and cancer, which are the biggest killers in developed nations.

The US Department of Agriculture (USDA) and Health and Human Services (HHS) believe that unhealthy eating is one of the leading causes of the obesity epidemic. The USDA and HHS recommend as a minimum 5 servings of fruits and vegetables, 2 servings of lean meat, and 6 servings of whole grains each day. They also suggest limiting saturated fats, added sugars, and sodium.

Adopting and maintaining a regular healthy eating plan will provide the following **BENEFITS** some time in the future:

- It will improve your heart's functioning, allowing it to effectively pump blood throughout your body
- It will allow you to get a good night's sleep, helping you feel energized during the day
- It will make you feel happier and increases your ability to cope with everyday stress
- It will keep your blood sugar levels in check, helping you lose weight.

Gain Abstract

The United States currently faces a growing public health concern with the obesity epidemic. Countless longitudinal studies have found a strong link between obesity and the development of chronic, non-communicable diseases such as heart disease, stroke, diabetes, and cancer, which are the biggest killers in developed nations.

The US Department of Agriculture (USDA) and Health and Human Services (HHS) believe that unhealthy eating is one of the leading causes of the obesity epidemic. The USDA and HHS recommend as a minimum 5 servings of fruits and vegetables, 2 servings of lean meat, and 6 servings of whole grains each day. They also suggest limiting saturated fats, added sugars, and sodium.

Adopting and maintaining a regular healthy eating plan will provide the following **BENEFITS** some time in the future:

- It will improve your cardiovascular health
- It will enhance your sleep
- It will improve your psychological well-being
- It will help you lose weight.

Loss Concrete

The United States currently faces a growing public health concern with the obesity epidemic. Countless longitudinal studies have found a strong link between obesity and the development of chronic, non-communicable diseases such as heart disease, stroke, diabetes, and cancer, which are the biggest killers in developed nations.

The US Department of Agriculture (USDA) and Health and Human Services (HHS) believe that unhealthy eating is one of the leading causes of the obesity epidemic. The USDA and HHS recommend as a minimum 5 servings of fruits and vegetables, 2 servings of lean meat, and 6 servings of whole grains each day. They also suggest limiting saturated fats, added sugars, and sodium.

Failing to adopt and maintain a regular healthy eating plan will lead to the following **RISKS** some time in the future:

- It will diminish your heart's functioning, preventing it from effectively pumping blood throughout your body
- It will disturb your sleep, making you feel tired and sluggish during the day
- It will cause you to feel more distressed and decreases your ability to cope with everyday stress
- It will increase your blood sugar levels in check, making you gain weight.

Loss Abstract

The United States currently faces a growing public health concern with the obesity epidemic. Countless longitudinal studies have found a strong link between obesity and the development of chronic, non-communicable diseases such as heart disease, stroke, diabetes, and cancer, which are the biggest killers in developed nations.

The US Department of Agriculture (USDA) and Health and Human Services (HHS) believe that unhealthy eating is one of the leading causes of the obesity epidemic. The USDA and HHS recommend as a minimum 5 servings of fruits and vegetables, 2 servings of lean meat, and 6 servings of whole grains each day. They also suggest limiting saturated fats, added sugars, and sodium.

Failing to adopt and maintain a regular healthy eating plan will lead to the following **RISKS** some time in the future:

- It will diminish your cardiovascular health
- It will disturb your sleep
- It will harm your psychological well-being
- It will make you gain weight

Gain Proximal

The United States currently faces a growing public health concern with the obesity epidemic. Countless longitudinal studies have found a strong link between obesity and the development of chronic, non-communicable diseases such as heart disease, stroke, diabetes, and cancer, which are the biggest killers in developed nations.

The US Department of Agriculture (USDA) and Health and Human Services (HHS) believe that unhealthy eating is one of the leading causes of the obesity epidemic. The USDA and HHS recommend as a minimum 5 servings of fruits and vegetables, 2 servings of lean meat, and 6 servings of whole grains each day. They also suggest limiting saturated fats, added sugars, and sodium.

Adopting and maintaining a regular healthy eating plan will lead to the following **IMMEDIATE BENEFITS** which may be noticeable within **WEEKS** of adherence:

- It will improve your heart's functioning
- It will help you sleep better
- It will put you in a good mood
- It will help you lose weight

Gain Distal

The United States currently faces a growing public health concern with the obesity epidemic. Countless longitudinal studies have found a strong link between obesity and the development of chronic, non-communicable diseases such as heart disease, stroke, diabetes, and cancer, which are the biggest killers in developed nations.

The US Department of Agriculture (USDA) and Health and Human Services (HHS) believe that unhealthy eating is one of the leading causes of the obesity epidemic. The USDA and HHS

recommend as minimums 5 servings of fruits and vegetables, 2 servings of lean meat, and 6 servings of whole grains each day. They also suggest limiting saturated fats, added sugars, and sodium.

Adopting and maintaining a regular healthy eating plan will lead to the following **LONG-TERM BENEFITS** which may be noticeable within **YEARS** of adherence:

- It will improve your heart's functioning
- It will help you sleep better
- It will put you in a good mood
- It will help you lose weight

Loss Proximal

The United States currently faces a growing public health concern with the obesity epidemic. Countless longitudinal studies have found a strong link between obesity and the development of chronic, non-communicable diseases such as heart disease, stroke, diabetes, and cancer, which are the biggest killers in developed nations.

The US Department of Agriculture (USDA) and Health and Human Services (HHS) believe that unhealthy eating is one of the leading causes of the obesity epidemic. The USDA and HHS recommend as minimums 5 servings of fruits and vegetables, 2 servings of lean meat, and 6 servings of whole grains each day. They also suggest limiting saturated fats, added sugars, and sodium.

Failing to adopt and maintain a regular healthy eating plan will lead to the following **IMMEDIATE RISKS**, which may be noticeable within **WEEKS** of inadherence:

- It will diminish your heart's functioning
- It will weaken your bones
- It will put you in a bad mood
- It will make you gain weight

Loss Distal

The United States currently faces a growing public health concern with the obesity epidemic. Countless longitudinal studies have found a strong link between obesity and the development of chronic, non-communicable diseases such as heart disease, stroke, diabetes, and cancer, which are the biggest killers in developed nations.

The US Department of Agriculture (USDA) and Health and Human Services (HHS) believe that unhealthy eating is one of the leading causes of the obesity epidemic. The USDA and HHS recommend as minimums 5 servings of fruits and vegetables, 2 servings of lean meat, and 6 servings of whole grains each day. They also suggest limiting saturated fats, added sugars, and sodium.

Failing to adopt and maintain a regular healthy eating plan will lead to the following **LONG-TERM RISKS**, which may be noticeable within **YEARS** of inadherence:

- It will diminish your heart's functioning
- It will weaken your bones
- It will put you in a bad mood
- It will make you gain weight

GOVERNMENT AGENCY: CDC

Gain Concrete

The United States currently faces a growing public health concern with the obesity epidemic. Countless longitudinal studies have found a strong link between obesity and the development of chronic, non-communicable diseases such as heart disease, stroke, diabetes, and cancer, which are the biggest killers in developed nations.

The Centers for Disease Control (CDC) believe that a lack of healthy eating is one of the leading causes of the obesity epidemic. The CDC recommends adults eat a variety of healthy, nutrient-dense foods across all food groups and set a calorie level that will help each individual achieve and maintain a healthy body.

Adopting and maintaining a regular healthy eating plan will provide the following **BENEFITS** some time in the future:

- It will improve your heart's functioning, allowing it to effectively pump blood throughout your body
- It will allow you to get a good night's sleep, helping you feel energized during the day
- It will make you feel happier and increases your ability to cope with everyday stress
- It will keep your blood sugar levels in check, helping you lose weight

Gain Abstract

The United States currently faces a growing public health concern with the obesity epidemic. Countless longitudinal studies have found a strong link between obesity and the development of chronic, non-communicable diseases such as heart disease, stroke, diabetes, and cancer, which are the biggest killers in developed nations.

The Centers for Disease Control (CDC) believe that a lack of healthy eating is one of the leading causes of the obesity epidemic. The CDC recommends adults eat a variety of healthy, nutrient-dense foods across all food groups and set a calorie level that will help each individual achieve and maintain a healthy body.

Adopting and maintaining a regular healthy eating plan will provide the following **BENEFITS** some time in the future:

- It will improve your cardiovascular health
- It will enhance your sleep
- It will improve your psychological well-being
- It will help you lose weight

Loss Concrete

The United States currently faces a growing public health concern with the obesity epidemic. Countless longitudinal studies have found a strong link between obesity and the development of chronic, non-communicable diseases such as heart disease, stroke, diabetes, and cancer, which are the biggest killers in developed nations.

The Centers for Disease Control (CDC) believe that a lack of healthy eating is one of the leading causes of the obesity epidemic. The CDC recommends adults eat a variety of healthy, nutrient-dense foods across all food groups and set a calorie level that will help each individual achieve and maintain a healthy body.

Failing to adopt and maintain a regular healthy eating plan will lead to the following **RISKS** some time in the future:

- It will diminish your heart's functioning, preventing it from effectively pumping blood throughout your body
- It will disturb your sleep, making you feel tired and sluggish during the day
- It will cause you to feel more distressed and decreases your ability to cope with everyday stress
- It will increase your blood sugar levels in check, leading you to gain weight

Loss Abstract

The United States currently faces a growing public health concern with the obesity epidemic. Countless longitudinal studies have found a strong link between obesity and the development of chronic, non-communicable diseases such as heart disease, stroke, diabetes, and cancer, which are the biggest killers in developed nations.

The Centers for Disease Control (CDC) believe that a lack of healthy eating is one of the leading causes of the obesity epidemic. The CDC recommends adults eat a variety of healthy, nutrient-dense foods across all food groups and set a calorie level that will help each individual achieve and maintain a healthy body.

Failing to adopt and maintain a regular healthy eating plan will lead to the following **RISKS** some time in the future:

- It will diminish your cardiovascular health
- It will disturb your sleep
- It will harm your psychological well-being
- It will lead you to gain weight

Gain Proximal

The United States currently faces a growing public health concern with the obesity epidemic. Countless longitudinal studies have found a strong link between obesity and the development of chronic, non-communicable diseases such as heart disease, stroke, diabetes, and cancer, which are the biggest killers in developed nations.

The Centers for Disease Control (CDC) believe that a lack of healthy eating is one of the leading causes of the obesity epidemic. The CDC recommends adults eat a variety of healthy, nutrient-dense foods across all food groups and set a calorie level that will help each individual achieve and maintain a healthy body.

Adopting and maintaining a regular healthy eating plan will lead to the following **IMMEDIATE BENEFITS** which may be noticeable within **WEEKS** of adherence:

- It will improve your heart's functioning
- It will help you sleep better
- It will put you in a good mood

- It will help you lose weight

Gain Distal

The United States currently faces a growing public health concern with the obesity epidemic. Countless longitudinal studies have found a strong link between obesity and the development of chronic, non-communicable diseases such as heart disease, stroke, diabetes, and cancer, which are the biggest killers in developed nations.

The Centers for Disease Control (CDC) believe that a lack of healthy eating is one of the leading causes of the obesity epidemic. The CDC recommends adults eat a variety of healthy, nutrient-dense foods across all food groups and set a calorie level that will help each individual achieve and maintain a healthy body.

Adopting and maintaining a regular healthy eating plan will lead to the following **LONG-TERM BENEFITS** which may be noticeable within **YEARS** of adherence:

- It will improve your heart's functioning
- It will help you sleep better
- It will put you in a good mood
- It will help you lose weight

Loss Proximal

The United States currently faces a growing public health concern with the obesity epidemic. Countless longitudinal studies have found a strong link between obesity and the development of chronic, non-communicable diseases such as heart disease, stroke, diabetes, and cancer, which are the biggest killers in developed nations.

The Centers for Disease Control (CDC) believe that a lack of healthy eating is one of the leading causes of the obesity epidemic. The CDC recommends adults eat a variety of healthy, nutrient-dense foods across all food groups and set a calorie level that will help each individual achieve and maintain a healthy body.

Failing to adopt and maintain a regular healthy eating plan will lead to the following **IMMEDIATE RISKS**, which may be noticeable within **WEEKS** of inadherence:

- It will diminish your heart's functioning
- It will impair your sleep
- It will put you in a bad mood
- It will make you gain weight

Loss Distal

The United States currently faces a growing public health concern with the obesity epidemic. Countless longitudinal studies have found a strong link between obesity and the development of chronic, non-communicable diseases such as heart disease, stroke, diabetes, and cancer, which are the biggest killers in developed nations.

The Centers for Disease Control (CDC) believe that a lack of healthy eating is one of the leading causes of the obesity epidemic. The CDC recommends adults eat a variety of healthy, nutrient-dense foods across all food groups and set a calorie level that will help each individual achieve and maintain a healthy body.

Failing to adopt and maintain a regular healthy eating plan will lead to the following **LONG-TERM RISKS**, which may be noticeable within **YEARS** of inadherence:

- It will diminish your heart's functioning
- It will impair your sleep
- It will put you in a bad mood
- It will make you gain weight

ADDITIONAL ANALYSES

Table S1. Mean reported perceived distance to when health consequences will be experienced.

					95% Confidence Interval	
**Construal Type**	**Level**	**Frame**	**Mean**	**SD**	**Lower Bound**	**Upper Bound**
Abstraction	Abstract	Gain	2.90	.917	2.769	3.037
		Loss	2.77	.871	2.642	2.902
	Concrete	Gain	2.77	1.02	2.644	2.902
		Loss	2.80	.971	2.674	2.931
Time	Distant	Gain	2.96	.953	2.828	3.086
		Loss	2.90	.1.03	2.768	3.032
	Proximal	Gain	2.56	.908	2.422	2.687
		Loss	2.27	.968	2.142	2.406

Table S2. Mean percent of healthy foods chosen (SD) when BMI, AGE, GENDER are not included.

	Frame	
**USDA and HHS recommendations**	**Gain**	**Loss**
Abstraction	68.24 (1.72)	74.20 (1.67)
Time	73.28 (1.72)	73.72 (1.73)
**CDC recommendations**	**Gain**	**Loss**
Abstraction	74.21 (1.66)	71.11 (1.66)
Time	70.63 (1.64)	73.80 (1.67)

Table S3. Model parameters when predicting percent of healthy options chosen in a model excluding covariates.

	Type III Sum of Squares	df	Mean Square	F	p	η_p^2
Construal Type	346.21	1	346.21	.59	.441	.000
Level	460.02	1	460.02	.79	.375	.000
Gain/Loss Frame	1075.03	1	1075.03	1.84	.175	.001
Government Agency	2.35	1	2.35	.004	.949	.000
Construal Type X Level	4169.89	1	4169.89	7.15	.008	.004
Construal Type X Gain/Loss Frame	14.40	1	14.40	.03	.875	.000
Construal Type X Government Agency	764.54	1	764.54	1.31	.252	.001
Level X Gain/Loss Frame	163.09	1	163.09	.28	.597	.000
Level X Government Agency	84.98	1	84.98	.15	.703	.000
Gain/Loss Frame X Government Agency	1027.60	1	1027.60	1.76	.184	.001
Construal Type X Level X Gain/Loss Frame	938.77	1	938.77	1.61	.205	.001
Construal Type X Level X Government Agency	508.93	1	508.93	.87	.350	.001
Construal Type X Gain/Loss Frame X Government Agency	3560.13	1	3560.13	6.11	.014	.004
Level X Gain/Loss Frame X Government Agency	420.74	1	420.74	.72	.396	.000
Construal Type X Level X Gain/Loss Frame X Government Agency	261.52	1	261.52	.45	.503	.000

Table S4. Model parameters when predicting percent of healthy options chosen in a model including covariates of age, gender, and BMI.

	Type III Sum of Squares	df	Mean Square	*F*	*p*	η_p^2
Age	29747.10	1	29747.10	53.47	.000	.032
Gender	7287.48	1	7287.48	13.10	.000	.008
BMI	9295.55	1	9295.55	16.71	.000	.010
Construal Type	124.40	1	124.40	.22	.636	.000
Level	676.86	1	676.86	1.22	.270	.001
Gain/Loss Frame	1271.11	1	1271.11	2.29	.131	.001
Government Agency	14.37	1	14.37	.03	.872	.000
Construal Type X Level	3826.22	1	3826.22	6.88	.009	.004
Construal Type X Gain/Loss Frame	17.51	1	17.51	.03	.859	.000
Construal Type X Government Agency	680.12	1	680.12	1.33	.269	.001
Level X Gain/Loss Frame	124.12	1	124.12	.22	.67	.000
Level X Government Agency	85.66	1	85.66	.15	.695	.000
Gain/Loss Frame X Government Agency	541.74	1	541.74	.97	.324	.001
Construal Type X Level X Gain/Loss Frame	1055.42	1	1055.42	1.90	.169	.001
Construal Type X Level X Government Agency	615.58	1	615.58	1.11	.293	.001
Construal Type X Gain/Loss Frame X Government Agency	3355.24	1	3355.24	6.03	.014	.004
Level X Gain/Loss Frame X Government Agency	.01	1	.01	.00	.997	.000
Construal Type X Level X Gain/Loss Frame X Government Agency	358.36	1	358.36	.64	.422	.000
